# D-limonene Inhibits Pentylenetetrazole-Induced Seizure via Adenosine A2A Receptor Modulation on GABAergic Neuronal Activity

**DOI:** 10.3390/ijms21239277

**Published:** 2020-12-04

**Authors:** Sowoon Seo, Yunjeong Song, Sun Mi Gu, Hyun Kyu Min, Jin Tae Hong, Hye Jin Cha, Jaesuk Yun

**Affiliations:** 1 College of Pharmacy and Medical Research Center, Chungbuk National University, Osongsaengmyeong 1-ro 194-31, Osong-eup, Heungduk-gu, Cheongju, Chungbuk 28160, Korea; olwg12@naver.com (S.S.); julietsyj@gmail.com (Y.S.); g09010327@nate.com (S.M.G.); gusrb4785@naver.com (H.K.M.); jinthong@chungbuk.ac.kr (J.T.H.); 2 Narcotics Policy Division, Ministry of Food and Drug Safety, Osong-eup, Heungduk-gu, Cheongju, Chungbuk 28160, Korea

**Keywords:** adenosine A2A receptor, convulsion, D-limonene, GABAergic neuron, kindling

## Abstract

Background: Epilepsy is a chronic neurological disorder characterized by the recurrence of seizures. One-third of patients with epilepsy may not respond to antiseizure drugs. Purpose: We aimed to examine whether D-limonene, a cyclic monoterpene, exhibited any antiseizure activity in the pentylenetetrazole (PTZ)-induced kindling mouse model and in vitro. Methods: PTZ kindling mouse model was established by administering PTZ (30 mg/kg) intraperitoneally to mice once every 48 h. We performed immunoblot blots, immunohistochemistry (IHC), and high-performance liquid chromatography (HPLC) analysis after the behavioral study. Results: An acute injection of PTZ (60 mg/kg) induced seizure in mice, while pretreatment with D-limonene inhibited PTZ-induced seizure. Repeated administration of PTZ (30 mg/kg) increased the seizure score gradually in mice, which was reduced in D-limonene (10 mg/kg)-pretreated group. In addition, D-limonene treatment increased glutamate decarboxylase-67 (GAD-67) expression in the hippocampus. Axonal sprouting of hippocampal neurons after kindling was inhibited by D-limonene pretreatment. Moreover, D-limonene reduced the expression levels of Neuronal PAS Domain Protein 4 (Npas4)-induced by PTZ. Furthermore, the adenosine A2A antagonist SCH58261 and ZM241385 inhibited anticonvulsant activity and gamma-aminobutyric acid (GABA)ergic neurotransmission-induced by D-limonene. Conclusion: These results suggest that D-limonene exhibits anticonvulsant activity through modulation of adenosine A2A receptors on GABAergic neuronal function.

## 1. Introduction

Approximately 70 million people have epilepsy worldwide. Antiseizure medications for epilepsy can be classified into several groups depending on the molecular targets, such as voltage-gated ion channels, gamma-aminobutyric acid inhibition, synaptic release machinery, and ionotropic glutamate receptors. However, approximately 30% of patients with epilepsy do not show response to any antiseizure medications [1]. Therefore, identification of new drug candidates is required for the protection against this chronic neurological disorder.

D-limonene is a common terpene found in citrus fruits. This monoterpene is widely used as a flavor and fragrance; it has been recognized as safe in food by the Food and Drug Administration [2]. D-limonene has been shown to exert anxiolytic effects, regulatory effects on neurotransmitters, and antinociceptive effects [3,4,5,6]. In addition, D-limonene has been reported to show effects on the synthesis/changes of neurotransmitters such as dopamine, serotonin, and GABA [6,7]. Furthermore, the effects of various essential oils on epilepsy and acute seizures were organized [8]. However, the potential of D-limonene in the treatment of epilepsy is still largely unknown.

Pentylenetetrazole-induced epilepsy model is widely used for the screening of anticonvulsant drugs. PTZ kindling model is used for the study of epileptogenesis process and discover new treatments [9]. In this study, we aimed to investigate the effect of D-limonene on PTZ-induced seizure and kindling in mice. In addition, to elucidate the possible mechanism underlying the effects of D-limonene in PTZ-induced seizure, we measured GABA, GAD-67, and Npas4 expression in the hippocampus of PTZ-induced kindled mice. D-limonene has been reported to be an agonist for adenosine A2A receptor [10], which is involved in convulsion-induced neurodegeneration [11]. Therefore, we examined whether adenosine A2A receptor antagonists inhibit the effects of D-limonene. SCH58261 and ZM241385 have a similar structure with adenine residue of adenosine and show adenosine A2A receptor antagonism [12,13]. Accordingly, we predicted that D-limonene has an anticonvulsant effect via adenosine A2A receptor modulation on GABAergic neuronal function.

## 2. Results

### 2.1. D-limonene Decreased PTZ-Induced Convulsion Dose Dependently

**Figure 1:**
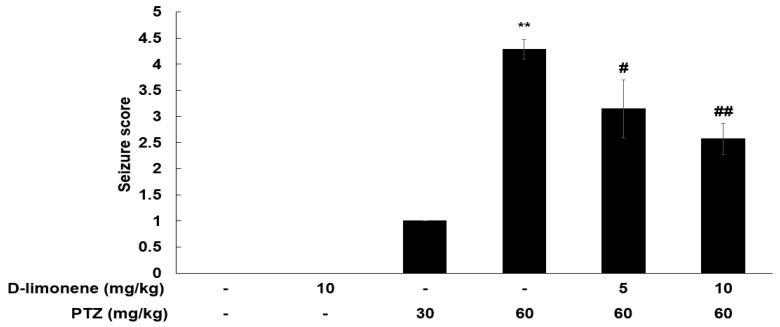
D-limonene dose-dependently inhibits PTZ-induced convulsion. Effect of D-limonene pretreatment on the development of PTZ-induced convulsion. Data are presented as the means ± S.E. (*n* = 6–7 for each group). ** *p* < 0.01 vs. vehicle (0.25% tween80 in saline) with saline, # *p* < 0.05 and ## *p* < 0.01 vs. vehicle (0.25% tween80 in saline) with PTZ 60 mg/kg (Kruskal–Wallis one-way ANOVA on ranks with Dunn’s post hoc analysis).

To assess the anticonvulsant activity of D-limonene, we investigated the effects of D-limonene on PTZ-induced convulsion. PTZ (60 mg/kg) injection induced significant seizures compared to the control (Figure 1). The mice were injected either vehicle (0.25% tween-80 in saline, 10 mL/kg) or acute dose D-limonene (5 or 10 mg/kg), 40 min before PTZ administration (60 mg/kg). D-limonene inhibited PTZ-induced seizure in a dose-dependent manner (Figure 1). In the PTZ treated group (60 mg/kg, *n* = 7), two mice showed a seizure score of 5, and five mice showed a score of 4. However, in D-limonene with PTZ treated group (10 mg/kg of D-limonene, 60 mg/kg of PTZ, *n* = 7), only one mouse showed seizure score of 4, while four mice and two others showed a seizure score of 2 and 3, respectively. These results suggest that D-limonene-treated mice showed a decrease in PTZ-induced convulsion.

### 2.2. D-limonene Inhibited the Development of PTZ-Induced Kindling

**Figure 2:**
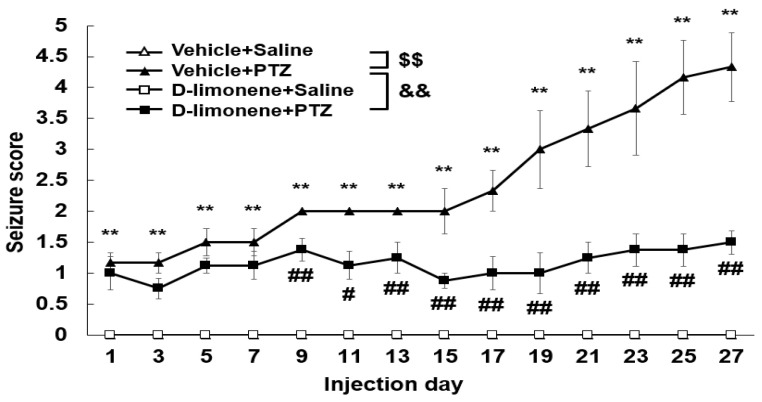
D-limonene inhibits PTZ induced kindling. Effect of D-limonene pretreatment on the development of PTZ-induced kindling. Data are presented as the means ± S.E. (*n* = 6~8 each group). ** *p* < 0.01 vs. vehicle (0.25% tween80 in saline) with saline, # *p* < 0.05 and ## *p* < 0.01 vs. vehicle (0.25% tween80 in saline) with PTZ, $$ *p* < 0.01, && *p* < 0.01 (Two-way RM ANOVA followed by Holm–Sidak test).

We investigated the effect of D-limonene on the development of PTZ-induced kindling. As shown in Figure 2, chronic administration of PTZ (30 mg/kg) induced kindling. However, D-limonene (10 mg/kg) pretreatment significantly decreased seizure scores. This finding revealed that D-limonene prevented PTZ-induced kindling.

### 2.3. D-limonene Inhibited Axonal Sprouting in the Hippocampus

**Figure 3:**
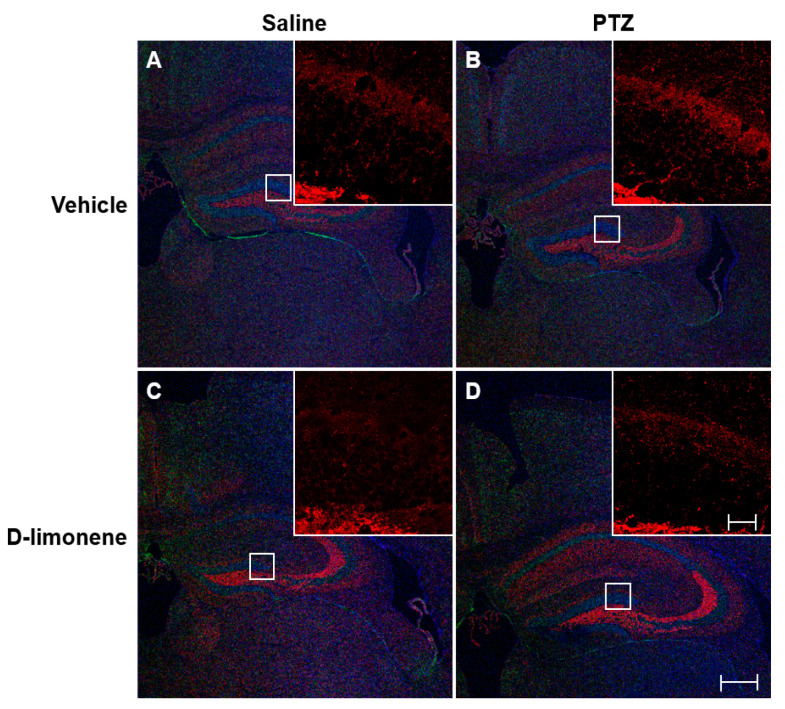
D-limonene inhibits PTZ induced mossy fiber sprouting. Representative sections of triple immunofluorescence staining with synaptoporin (red), NeuN (green), DAPI (blue). (**A**). vehicle (0.25% tween80 in saline) and saline treated group; (**B**). vehicle (0.25% tween80 in saline) and PTZ treated group; (**C**). D-limonene and saline treated group; (**D**). D-limonene and PTZ treated group. Large scale bar represents 100 µm; small scale bar represents 10 µm.

To investigate the changes in PTZ-induced axonal sprouting in the hippocampus, caused by D-limonene treatment, the mice were immediately sacrificed after scoring the seizures on the last day of kindling. Then, IHC was performed. The increase in axonal sprouting induced by PTZ (Figure 3B) was reduced by D-limonene (Figure 3D). Mossy fiber sprouting also occurred in CA3 and decreased by D-limonene (Figure S1). These data showed that D-limonene could prevent PTZ-induced axonal sprouting.

### 2.4. D-limonene Regulated GAD-67 and Npas4 Expression Levels

**Figure 4:**
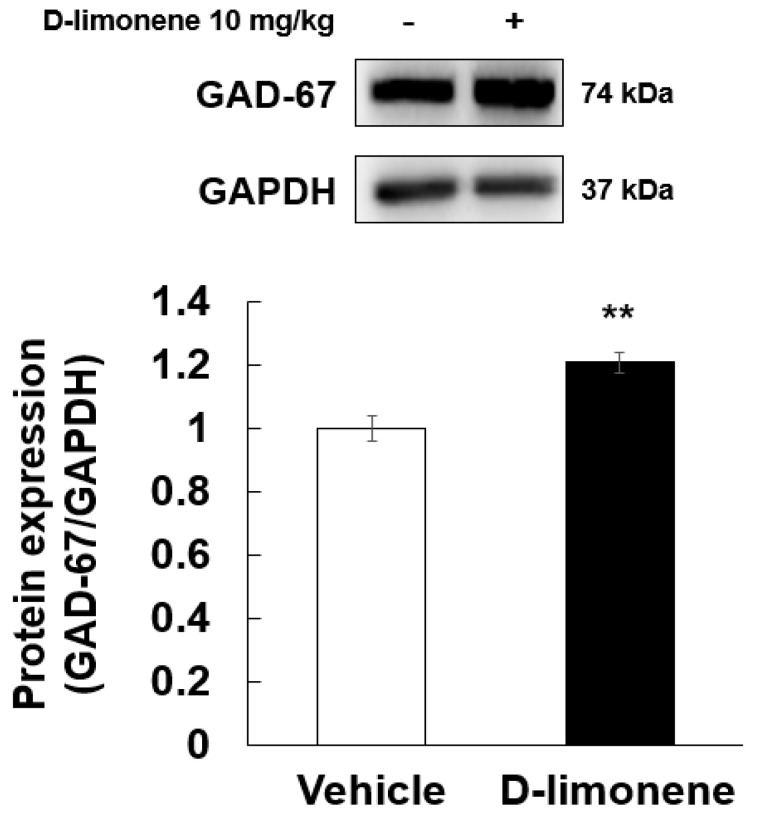
D-limonene significantly increased the expression of GAD-67 proteins. Relative protein expression level of GAD-67/GAPDH. Data are presented as the means ± S.E. (*n* = 4 for each group). ** *p* < 0.01 vs. vehicle (0.25% tween80 in saline) (Student’s t-test).

**Figure 5:**
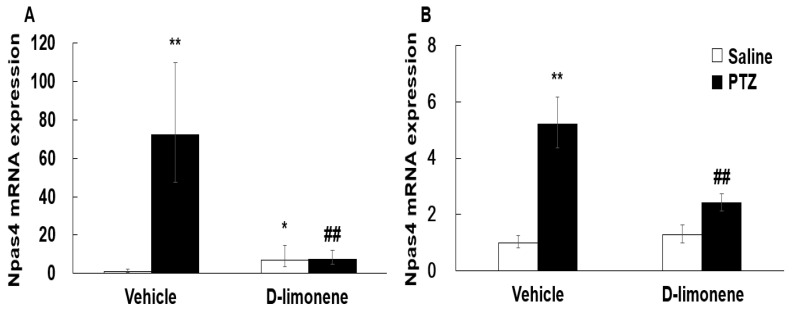
D-limonene inhibited PTZ induced Npas4 mRNA expression levels. (**A**). Npas4 mRNA expression level after acute injection of D-limonene and PTZ. (**B**) Npas4 mRNA expression level after chronic injection (in kindling experiments) of D-limonene and PTZ. Data are presented as the means ± S.E. (*n* = 4–6 for each group). * *p* < 0.05, ** *p* < 0.01 vs. vehicle (0.25% tween80 in saline) with saline, ## *p* < 0.01 vs. vehicle (0.25% tween80 in saline) with PTZ (Two-way ANOVA followed by Holm–Sidak test).

According to a previous result [6], D-limonene increased GABA level in the whole brain. Therefore, to elucidate the possible mechanism underlying the effect of D-limonene on seizures, we investigated the expression levels of GABA synthesis enzyme (GAD-67) by Western blotting analysis. Our Western blotting data showed that D-limonene treatment increased the expression of GAD-67 protein (Figure 4, Figure S2). We further investigated whether D-limonene regulates transcription factors such as Npas4, which is induced by neuronal activity. Both acute and chronic administration of PTZ induced an increase in Npas4 expression in the hippocampus, which was inhibited by D-limonene (Figure 5). D-limonene reduced convulsion induced by PTZ; therefore, we assume thatNpas4 expression by PTZ was reduced by D-limonene.

### 2.5. D-limonene Modulated GABA Levels in the Hippocampus and PC12 Cells

**Figure 6:**
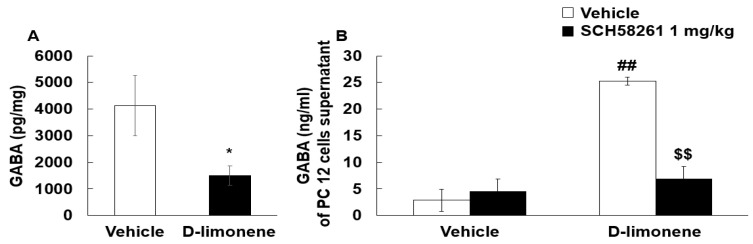
D-limonene decreased GABA level in the hippocampus and increased GABA release in PC12 cells. (**A**). Hippocampal GABA levels assessed by HPLC are shown for vehicle (0.25% tween80 in saline) and D-limonene treated mice. Data are presented as the means ± S.E. (*n* = 7 for each group) * *p* < 0.05 vs. vehicle (0.25% tween80 in saline) (Student’s t-test). (**B**). GABA release according to D-limonene treatment in PC12 cells. Data are presented as the means ± S.E. (*n* = 4–8 for each group). ## *p* < 0.01 vs. vehicle (0.25% tween80 in saline) with vehicle (DMSO:tween80:saline = 1:1:18), $$ *p* < 0.01 vs. D-limonene with vehicle (DMSO:tween80:saline = 1:1:18) (Two-way ANOVA followed by Holm–Sidak test).

The inhibitory neurotransmitter GABA may play a role in the anticonvulsant effects of D-limonene. Therefore, we examined whether D-limonene treatment could have effects on GABA levels in the hippocampus. Surprisingly, the mice treated with acute D-limonene showed a decrease in GABA levels in the hippocampus compared with the vehicle (0.25% tween-80 in saline, 10 mL/kg)-treated group (Figure 6A). We further investigated the effect of D-limonene on GABAergic neurotransmission and the underlying mechanism in PC12 cells. GABA levels in culture media of PC12 cells were enhanced by D-limonene pretreatment and reduced by SCH58261 (Figure 6B). These results suggest that D-limonene regulates GABAergic neurotransmission.

### 2.6. D-limonene Suppresses Seizure Score Induced by PTZ via Adenosine A2A Receptor

**Figure 7:**
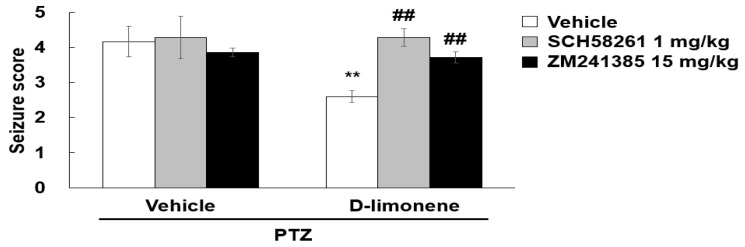
Adenosine A2A receptor antagonist restores the seizure score suppressed by D-limonene. Effects of D-limonene on PTZ-induced convulsion exerted by binding to adenosine A2A receptor. There was no change in a seizure score of 0 on average in the saline-treated group compared to the PTZ-treated group. Data are presented as the means ± S.E. (*n* = 6–8 for each group). ** *p* < 0.01 vs. vehicle (0.25% tween80 in saline) with Vehicle (DMSO:tween80:saline = 1:1:18), ## *p* < 0.01 vs. D-limonene with vehicle (DMSO:tween80:saline = 1:1:18) (Two-way ANOVA followed by Holm-Sidak test).

To investigate whether D-limonene decreased the seizure score induced by PTZ via adenosine A2A receptor, SCH58261, or ZM241385 was treated prior to D-limonene injection, and a convulsion behavioral test was performed. We found that the seizure score suppressed by D-limonene was recovered by pretreatment with SCH58261 or ZM241385 (Figure 7). However, treatment of SCH58261 or ZM241385 with vehicle (0.25% tween 80 in saline) or D-limonene did not induce convulsion (data not shown). This finding revealed that D-limonene may inhibit PTZ-induced convulsion via the modulation of adenosine A2A receptor activation.

### 2.7. D-limonene Enhanced Phosphorylated cAMP Response Element-Binding Protein in the Hippocampus

**Figure 8:**
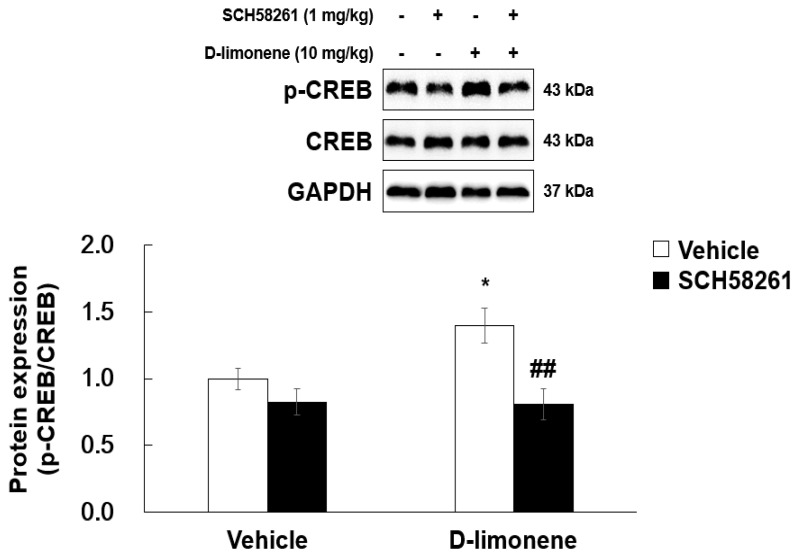
D-limonene enhances phosphorylated form of CREB in the hippocampus via adenosine A2A receptor activation. Relative protein expression level of p-CREB/CREB. Data are presented as the means ± S.E. (*n* = 4 for each group). * *p* < 0.05 vs. vehicle (0.25% tween80 in saline) with Vehicle (DMSO:tween80:saline = 1:1:18), ## *p* < 0.01 vs. D-limonene with vehicle (DMSO:tween80:saline = 1:1:18) (Two-way ANOVA followed by Holm–Sidak test).

As D-limonene is an adenosine A2A receptor agonist, we investigated whether D-limonene has an effect on phosphorylated cAMP response element-binding (phospho-CREB) protein levels in the adenosine A2A receptor downstream signaling pathway. Acute injection of D-limonene (10 mg/kg) increased phospho-CREB levels in the hippocampus. However, total CREB expression remained unchanged. Furthermore, SCH58261 pretreatment reduced D-limonene-induced phospho-CREB upregulation (Figure 8).

## 3. Discussion

In this study, we aimed to investigate the anti-convulsant effects of D-limonene on PTZ-induced seizure and kindling in mice. We demonstrated that D-limonene reduced seizure scores in a dose-dependent manner and significantly decreased seizure severity in PTZ-kindled mice. D-limonene maintained a seizure score of 1.15 on average until the last day of PTZ kindling, whereas mice in the vehicle (0.25%, tween-80 in saline, 10 mL/kg) group were fully kindled.

Mossy fiber sprouting is a histological change that is typically observed in the brain of patients with temporal lobe epilepsy [14,15]. Animal convulsion models showed similar changes in the hippocampus [16]. We demonstrated that PTZ kindling induced axonal sprouting in the hippocampus, and these histological changes were reduced by D-limonene pretreatment.

In previous studies, it was suggested that s-limonene may activate the GABAergic system and (+)-limonene epoxide inhibit convulsion induced by picrotoxin or PTZ through the regulation GABAergic system [6,17]. We observed that GABA levels in culture media of PC12 cells increased following treatment with D-limonene. We assume that this upregulation of GABA is due to agonist activity of D-limonene on adenosine A2A receptor, which increased the GABA release [18]. However, tissue GABA level was reduced by D-limonene. This lowed GABA level may be associated with the increased GAD-67 expression and GABA turnover.

Furthermore, the increase of GABA levels in PC12 cells was attenuated by the treatment with SCH58261, an adenosine A2A receptor antagonist. Adenosine A2A receptor is expressed in GABAergic neurons and enhances GABA synaptic transmission in the hippocampus [19]. In addition, adenosine A2A receptors induce excitatory neuronal activity via glutamate neurotransmission [18,20]. These results suggest that D-limonene may increase GABA release via glutamate outflow in the hippocampus.

The brain-specific transcription factor Npas4 is known to play a role in the development of inhibitory synapses, and its expression is regulated by neuronal activity [21]. Npas4 also plays a role in regulating the inhibitory–excitatory balance [22]. In the present study, PTZ increased Npas4 mRNA levels; however, these levels were reduced by both acute and chronic injection of D-limonene. D-limonene may reduce Npas4 expression via the reduction of convulsion induced by PTZ. However, D-limonene itself increased Npas4 expression. Additional studies are needed to further elucidate the mechanism underlying Npas4 regulation of D-limonene.

Adenosine A2A receptor stimulation induced activation of cell signaling such as cyclic adenosine monophosphate (cAMP) protein kinase A and phosphorylation of CREB [23]. It has been demonstrated that the concentration of cAMP increased when CHO cells transfected with adenosine A2A receptor were treated with D-limonene [10]. In the present study, we showed that phospho-CREB levels induced by adenosine A2A receptor downstream signaling were increased by D-limonene and decreased by pretreatment with SCH58261 in the hippocampus. In addition, seizures induced by PTZ were prevented by D-limonene. However, pretreatment with SCH58261 or ZM241385 inhibited the anti- convulsant activity of D-limonene.

Taken together, it can be suggested that D-limonene exerts anticonvulsant effects via the regulation on adenosine A2A receptor activation and GABAergic neurotransmission.

## 4. Materials and Methods

### 4.1. Drugs

R-(+)-Limonene (C10H16) of 97% purity was purchased from Sigma-Aldrich (St. Louis, MO, USA) and prepared every day as a fresh solution by dissolving in 0.25% tween-80 in saline. SCH58261 was purchased from Tocris (Bristol, UK). PTZ, ZM241385 and other reagents were purchased form Sigma-Aldrich unless otherwise stated.

### 4.2. Animals

C57BL/6J mice were purchased from Daehan Bio Link (Chungbuk, Korea). The animals at 8–11 weeks old were maintained at 4–5 per cage under standard conditions (23 ± 2 °C, 50 ± 5% humidity) with a controlled 12 h light/dark cycle. Drinking water and rodent chow were provided ad libitum. All mice weighed 20–25 g at the beginning of the experiment. All the experiments were carried out according to the Guidelines for the care and use of animals (Chungbuk National University).

### 4.3. Cell Culture

PC12 cells, a cell line derived from the pheochromocytoma of rat adrenal medulla, were procured from Korean Collection for Type Cultures. The cells were cultured in standard conditions in RPMI cell culture medium supplemented with fetal bovine serum (FBS, 5%), Horse serum (10%, Gibco, Grand Island, USA), and antibiotic (1%, Gibco) under CO2 (5%) and high atmospheric humidity at 37 °C. For this study, cells on Poly-L-Lysine-coated 35 mm plates at passage 4–6 were used, and the cells were seeded at a density of 1 × 106 cells/plate.

### 4.4. Pentylenetetrazole-Induced Convulsion and Kindling Model

The mice were administered PTZ intraperitoneally (i.p.) at a dose of 30 or 60 mg/kg, and the intensity of convulsions induced by PTZ was scored according to previous studies [24]. The convulsion behaviors were observed for 30 min. The seizures were classified as follows: stage 0, normal behavior, no abnormality; 1: immobilization or lying on belly; 2: head nodding and facial, forelimb, or hindlimb myoclonus; 3: continuous whole-body myoclonus, myoclonic jerks or tail held up stiffly; 4: rearing, tonic seizure or falling down on its side. 5: tonic-clonic seizure, falling down on its back, wild rushing or jumping; 6: death [25] For establishing the mouse model of PTZ-induced kindling, 30 mg/kg PTZ was administered intraperitoneally once every 48 h (total 14 times). The mice showing more than three consecutive stage 4 seizure levels were defined as kindled mice. Brain samples for immunoblotting, IHC, HPLC, and RT-PCR were obtained immediately after final behavioral tests.

### 4.5. Effects of D-limonene on PTZ-Induced Convulsion and Kindling

To study the effects of D-limonene on PTZ-induced convulsion, D-limonene (5 or 10 mg/kg, i.p.) was administered intraperitoneally 40 min before PTZ injection (60 mg/kg). We measured seizure score for 30 min. For evaluating the effects of D-limonene on PTZ-induced kindling, the mice were pretreated with D-limonene (10 mg/kg) 40 min before injection of PTZ (30 mg/kg). Convulsion behaviors were observed for 30 min. To examine the role of adenosine A2A receptor in D-limonene-induced anticonvulsant activity, the mice were treated with SCH58261 (1 mg/kg, i.p.) or ZM241385 (15 mg/kg, i.p.) 10 min prior to D-limonene injection. At 40 min after D-limonene injection, PTZ-induced convulsion behavior test was performed.

### 4.6. Immunoblotting

Hippocampal homogenates were determined using Bio-Rad DC protein assay kit (BIO-RAD, Hercules, CA, USA) according to the manufacturer’s protocol. For immunoblotting analysis, 30 μg hippocampus proteins were resolved over 12% Tris-glycine polyacrylamide gel and then transferred onto polyvinylidene fluoride membrane (Merck Millipore, Burlington, VT, USA). The blots were blocked using 5% nonfat dry milk, and these immunoblots were probed using the primary antibodies Tyrosine hydroxylase (EMD Millipore, Darmstadt, Germany), GAD-67 (Abcam, Cambridge, UK), CREB (Cell signaling, Danvers, MA, USA), phospho-CREB (EMD Millipore), GAPDH (Cell signaling) and secondary horseradish peroxidase conjugate (Amersham Life Science Inc., IL, USA). The proteins were detected by chemiluminescence using the ECL kit (Amersham Life Science). The relative density of the protein bands was scanned and quantified by ImageJ (Wayne Rasband, National Institutes of Health, Bethesda, MD, USA).

### 4.7. RNA Extraction and Real-Time RT-PCR

RNA from frozen hippocampus was extracted using QIAGEN RNA kit (QIAGEN, Hilden, Germany), and cDNA was synthesized from 1 μg total RNA using a high-capacity cDNA reverse transcription kit (Applied Biosystems, Foster city, CA, USA). The amplification reactions were performed with 12.5 μL 2 × SYBR Green PCR master mix (Applied Biosystems), 1 μg cDNA, and forward and reverse primers (100 μM each) using StepOne Plus real-time RCR system (Applied Biosystems). The cycling parameters were as follows: 50 °C for 2 min and 95 °C for 10 min; 1 cycle of 95 °C for 15 s and 40 cycles of 60 °C for 2 min, after one initial step at 95 °C for 30 s and 55 °C for 30 s finally. The primers used were as follows: mouse Npas4, forward 5′-AGCATTCCAGGCTCATCTGAA-3′ and reverse 5′-GGCGAAGTAAGTCTTGGTAGGATT-3′; mouse GAPDH, forward 5′-TGTCAAGCTCATTTCCTGGTATGA-3′ and reverse 5′-TCTTACTCCTTGGAGGCCATGTAG-3′.

### 4.8. Immunohistochemistry

The mice were anesthetized and transcardially perfused with cold phosphate buffered saline (PBS, with 0.1% heparin) prior to a cold 4% paraformaldehyde (PFA) in PBS. The brains were post-fixed overnight with 4% PFA in PBS at 4 °C and then coronally cut with a Leica CM1850 microtome (10 µm thick sections). The sections were then permeabilized in PBS with 0.3% Triton X-100 and 0.3% hydrogen peroxide and blocked in 5% bovine serum albumin (BSA, with 1% goat serum) for 1 h at room temperature (RT). Then, the sections were incubated with primary antibodies diluted in 5% BSA at 4 °C overnight using rabbit Synaptoporin (SynapticSystems, 1/300) and mouse-NeuN (EMD Millipore, 1/500). After rinsing three times in PBS, the slices were incubated with the corresponding Alexa Fluor-conjugated secondary antibodies diluted in 5% BSA for 1 h at RT and finally stained for 20 min with DAPI (300 nM in 5% BSA, Sigma-Aldrich). The sections were then mounted onto glass slides (Matsunami, Osaka, Japan) with mounting medium (Vector Laboratories, Inc., San Francisco, CA, USA). For each section, images were taken using a 2.5X or 40X objective using a confocal microscope (LSM-880, ZEISS, Oberkochen, Germany) setting.

### 4.9. High-Performance Liquid Chromatography Analysis

After the final behavioral analysis, the mice were killed immediately. The brains were then removed, and the hippocampus was separated on an ice-chilled glass plate. These tissues were quickly frozen on dry ice and stored at −80 °C until assay. The frozen tissues were weighed and placed in 1.5 mL Eppendorf tubes and homogenized in 1 mL ice-cold PBS. For PC12 cells, the control groups were cultured with complete media, the limonene group was treated with limonene (30 µM) for 1 h, SCH58261 group was treated with SCH58261 (1 µM) for 30 min, and SCH58261+limonene group was treated with SCH58261 (1 µM) for 30 min, followed by treatment with limonene (30 µM) for 1 h, and the supernatants were obtained. After the centrifugation at 20,000 G for 15 min at 4 °C, clear supernatants were filtered through 0.22 μm CHROMDISC syringe filter. For detection of GABA, 180 μL of sample was added to 10 μL of reactive solution prepared from o-phthaldialdehyde (37 mM) in 0.1 M borate buffer (pH 10.4) containing 250 μL ethanol and 50 mM sodium sulfite. A UK-C18 column (50 × 2 mm, 3 μm; Imtakt) was coupled with an electrochemical detector (Eicom) with a glassy carbon electrode set at 0.85 V. The mobile phase comprised 50 mM phosphoric acid, 50 mM citric acid, 0.1 mM EDTA (pH 3.5), and 5% acetonitrile filtered through 0.45-μm filters (EMD Millipore) at a flow rate of 0.3 mL/min. The samples (20 μL) were then injected into the HPLC system (Waters Corporation, Milford, MA, USA).

### 4.10. Data Analysis

The data represent the mean ± SE. Data were analyzed using a Student’s t-test, one-way, two-way, or two-way repeated measures [13] analysis of variance (ANOVA), followed by Holm–Sidak post-hoc tests using SigmaPlot 13 software (SigmaPlot, Chicago, IL, USA). Non-parametric data were analyzed using a Kruskal Wallis ANOVA on ranks followed by Dunn’s post hoc analysis using SigmaPlot 13 software.

## Supplementary Materials

Supplementary materials can be found at https://www.mdpi.com/1422-0067/21/23/9277/s1.

## Abbreviations

CREB	cAMP response element-binding protein
GABA	Gamma-aminobutyric acid
GAD-67	Glutamate decarboxylase-67
HPLC	High-performance liquid chromatography
Npas4 PC12 cell PTZ	Neuronal PAS Domain Protein 4 Pheochromocytoma cells derived from the adrenal gland of Rattus norvegicus Pentylenetetrazole
