# Supplementary material for: D-limonene Inhibits Pentylenetetrazole-Induced Seizure via Adenosine A2A Receptor Modulation on GABAergic Neuronal Activity
Source: Int J Mol Sci. 2020 Dec 4;21(23):9277. doi: 10.3390/ijms21239277 (PMC7730947; doi:10.3390/ijms21239277)
Supplement: Supplementary file 1 [file ijms-21-09277-s001.pdf]

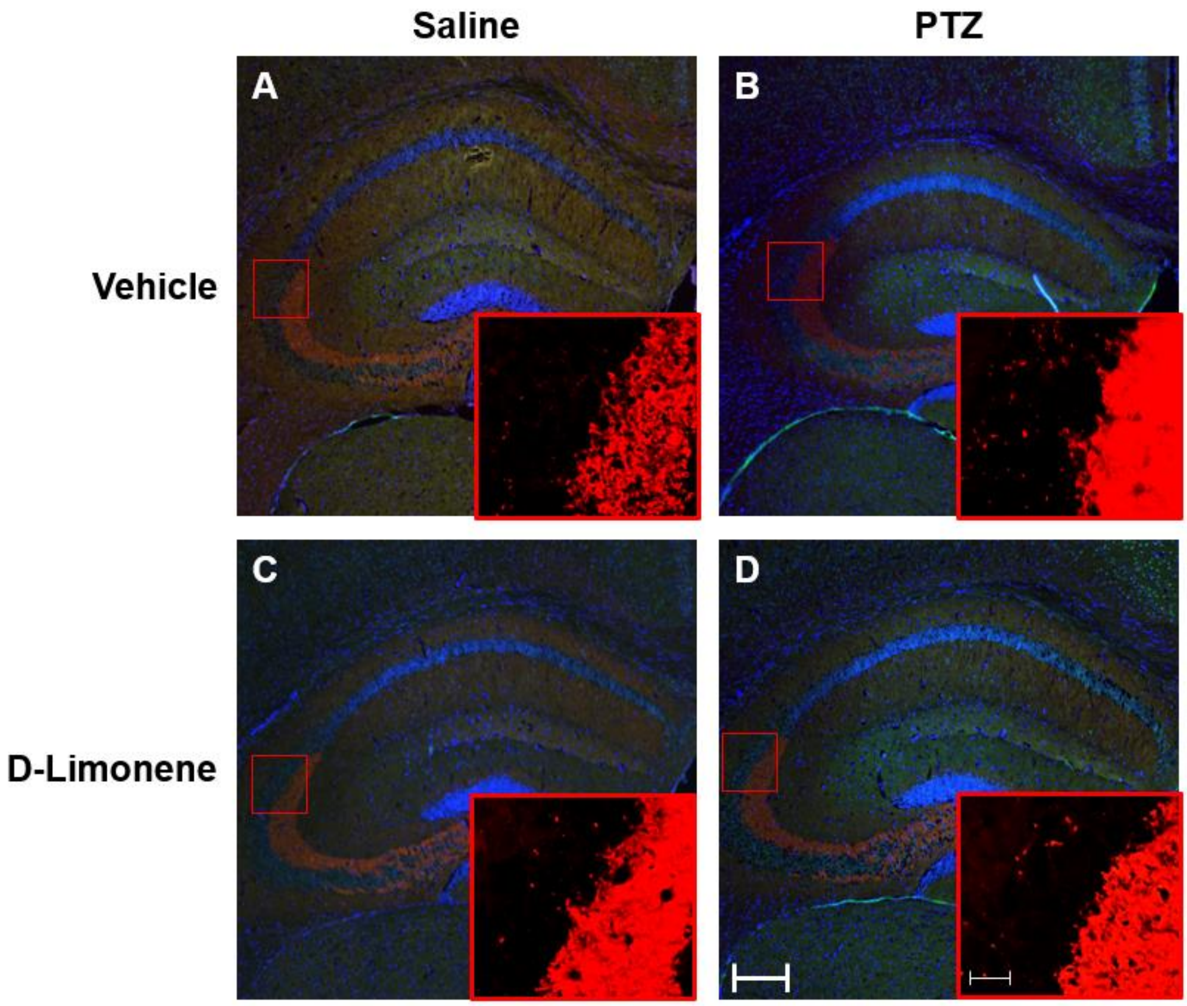


**Supplement 1.** D-limonene inhibits PTZ induced mossy fiber sprouting. Representative sections of triple immunofluorescence staining with synaptoporin (red), NeuN (green), DAPI (blue) in CA3. A. vehicle (0.25% tween80 in saline) and saline treated group; B. vehicle (0.25% tween80 in saline) and PTZ treated group; C. D-limonene and saline treated group; D. D-limonene and PTZ treated group. Large scale bar represents 50 μm; Small scale bar represents 10 μm

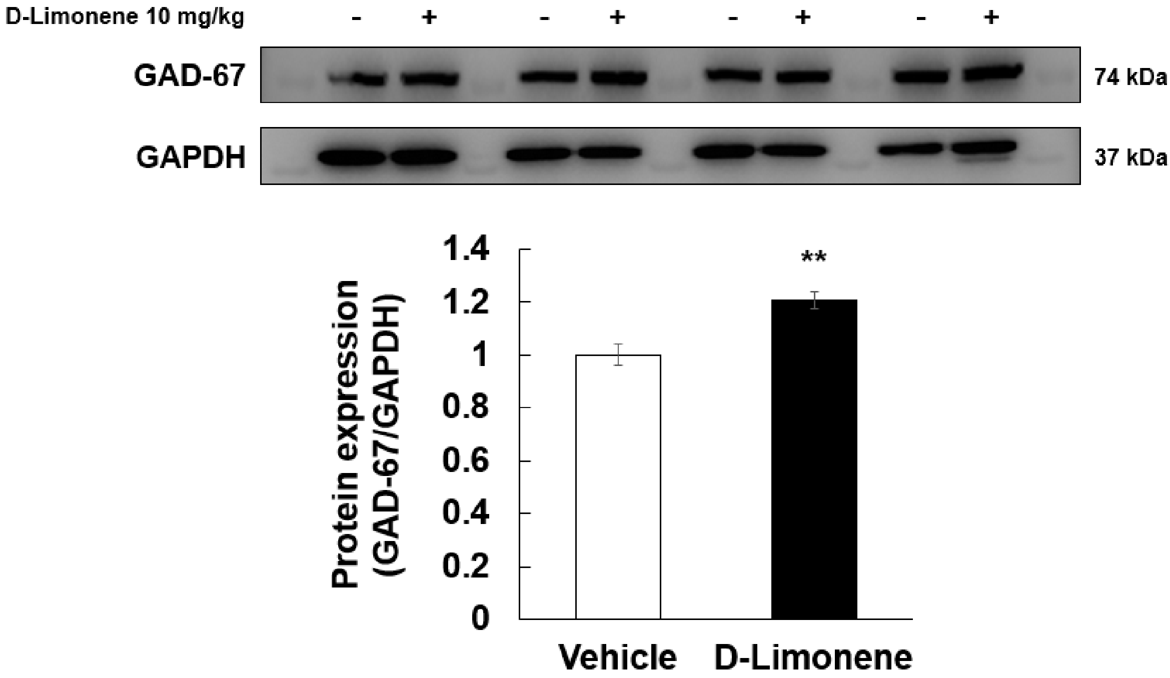


**Supplement 2.** D-limonene significantly increased the expression of GAD-67 proteins. Relative protein expression level of GAD-67/GAPDH. Data are presented as the means ± S.E. (n=4 for each group). * $p < 0.05$ vs Saline-Vehicle (0.25% tween80 in saline) (Student's t-test).
